# Political prioritization for digital health and health equity through global health diplomacy

**DOI:** 10.34172/hpp.43067

**Published:** 2024-07-29

**Authors:** Vijay Kumar Chattu, Sujatha Alla, Bawa Singh

**Affiliations:** ^1^Center for Global Health Research, Saveetha Medical College and Hospitals, Saveetha Institute of Medical and Technical Sciences (SIMATS), Saveetha University, Chennai, India; ^2^ReSTORE lab, Department of OS & OT, Temerty Faculty of Medicine, University of Toronto, Toronto, ON M5G 1V7, Canada; ^3^Department of Community Medicine, Faculty of Medicine, Datta Meghe Institute of Medical Sciences (DMIMS), Wardha, India; ^4^Department of Engineering Management & Systems Engineering, Frank Batten College of Engineering, Old Dominion University, Norfolk, VA 23529, USA; ^5^Center for Evidence-based Diplomacy, Global Health Research and Innovations Canada (GHRIC), Toronto, ON M1J 2W8, Canada; ^6^Department of South and Central Asian Studies, School of International Studies, Central University of Punjab, Bathinda, India

 We have found the article “Digital health and health equity: How digital health can address healthcare disparities and improve access to quality care in Africa” published in the recent issue to be very relevant and interesting.^1^ Qoseem et al have highlighted that there is a great need for continuous advocacy for strengthening the health systems through telehealth promotion and increasing access to healthcare services to achieve health equity.^1^

 As researchers in global health, technology, and politics, we acknowledge the fact that health equity and access to health care and medicines are the keys to a country as they impact well-being, quality of life, mortality rates, and overall development. However, these aspects of addressing disparities need a strong political will and must be prioritized in the national agendas. Therefore, we want to highlight that beyond advocacy, there is a great need for global health diplomacy (GHD) which can be a catalyst in addressing the root causes of inequities resulting in successful policies and resolutions to address equity such as Universal Health Coverage, Framework Convention of Tobacco Control, WHO’s International Health Regulations^2^ and African Medicines Agency^3^ to name a few. As highlighted by Chattu et al, GHD can be useful in addressing the disparities and improving access to medicines.^4^

 The authors also concluded that there is a need for international collaborations and investments in health interventions which is again very important to fill the inequality gaps and scale up the digital health tools. In this context, we stress that only through GHD we can strengthen the global partnerships and negotiate for funding mechanisms from the developed countries and other multilateral organizations. The other barrier we want to highlight is the role of Intellectual Property Rights (IPR) which hampers access to medicines, therapeutics, and diagnostics due to the patent issues. However, GHD played a significant role in negotiating for the TRIPS waiver for the COVID-19 vaccines and medicines and also in the establishment of the COVID-19 Vaccines Global Access (COVAX) facility.^5^ We conclude that GHD is an effective strategy that can bring in multiple partners from multiple sectors at multiple levels globally with a common agenda to take appropriate measures to address the gaps and disparities. Unless there is political prioritization, the journey of achieving equity and improving access remains a dream.

## Competing Interests

 None declared.

## Ethical Approval

 Not applicable.
